# Bayesian Modeling and Chronological Precision for Polynesian Settlement of Tonga

**DOI:** 10.1371/journal.pone.0120795

**Published:** 2015-03-23

**Authors:** David Burley, Kevan Edinborough, Marshall Weisler, Jian-xin Zhao

**Affiliations:** 1 Department of Archaeology, Simon Fraser University, Burnaby, BC, Canada; 2 Institute of Archaeology University College London, London, United Kingdom; 3 School of Social Science, University of Queensland, St. Lucia, Queensland, Australia; 4 School of Earth Sciences, University of Queensland, St. Lucia, Queensland, Australia; ICREA at the Universitat Autònoma de Barcelona, SPAIN

## Abstract

First settlement of Polynesia, and population expansion throughout the ancestral Polynesian homeland are foundation events for global history. A precise chronology is paramount to informed archaeological interpretation of these events and their consequences. Recently applied chronometric hygiene protocols excluding radiocarbon dates on wood charcoal without species identification all but eliminates this chronology as it has been built for the Kingdom of Tonga, the initial islands to be settled in Polynesia. In this paper we re-examine and redevelop this chronology through application of Bayesian models to the questioned suite of radiocarbon dates, but also incorporating short-lived wood charcoal dates from archived samples and high precision U/Th dates on coral artifacts. These models provide generation level precision allowing us to track population migration from first Lapita occupation on the island of Tongatapu through Tonga’s central and northern island groups. They further illustrate an exceptionally short duration for the initial colonizing Lapita phase and a somewhat abrupt transition to ancestral Polynesian society as it is currently defined.

## Introduction

Willard Libby’s 1949 development of radiocarbon dating is arguably the single greatest scientific advancement in the history of archaeology. Providing absolute dates for a wide range of material, it underlies the establishment of refined archaeological chronologies throughout the world. Over the past 65 years, there have been many advances in measurement techniques, in our abilities to calibrate radiocarbon years into calendar years, in the protocols accepted for sample selection, and in understanding more fully the relationship between chronometric dates and archaeological events of interest [1], [2]. At least a few can be called revolutionary, as they have reset chronological knowledge with substantive consequence. One of these revolutions currently is underway in Polynesia, where prehistory is of short duration and, by world standards, relatively recent. Consequently there is increasing concern for chronological accuracy and precision through exclusive dating of short-lived samples from well-defined archaeological contexts. The consequences are most apparent for East Polynesia, resulting in substantially shortened sequences for human presence and subsequent cultural development [3–6]. Elsewhere, to the west, island chronologies are also being questioned because they largely are built on unidentified wood charcoal samples with possibilities for inbuilt age, or shell dates where marine reservoir offsets and related issues are unresolved. A complete reevaluation of the entire chronology for Oceanic settlement has been advocated [7].

Tonga is positioned on the western flank of the Polynesian triangle, and it was the first island group to be settled in Polynesia [8]. A previous PlosOne paper [9] establishes a secure beginning point for the settlement of Tonga, providing high precision U/Th dates on coral artifacts for the founder colony of Nukuleka. A similarly precise chronology for Polynesian expansion and associated events throughout Tonga has yet to be addressed. This is equally critical for it is here that ancestral Polynesian society emerges [10]. Previous excavations at multiple sites in the Kingdom provide numerous radiocarbon dates, virtually all with secure stratigraphic and archaeological associations [11–13]. Contemporary protocols for chronometric hygiene requiring samples to be identified short-lived wood species, nuts, or twigs purge all but a few [14]. In this paper we redevelop this chronology employing Bayesian statistical models integrating existing dates with newly identified short-lived sample radiocarbon dates and additional high precision U/Th dates on coral artifacts. Our results provide generation-type precision going beyond the constraints of radiocarbon measurement alone. This facilitates new insight into Tongan settlement and the rapidity with which ancestral Polynesian society emerges as it is now defined.

## Context

**Fig 1:**
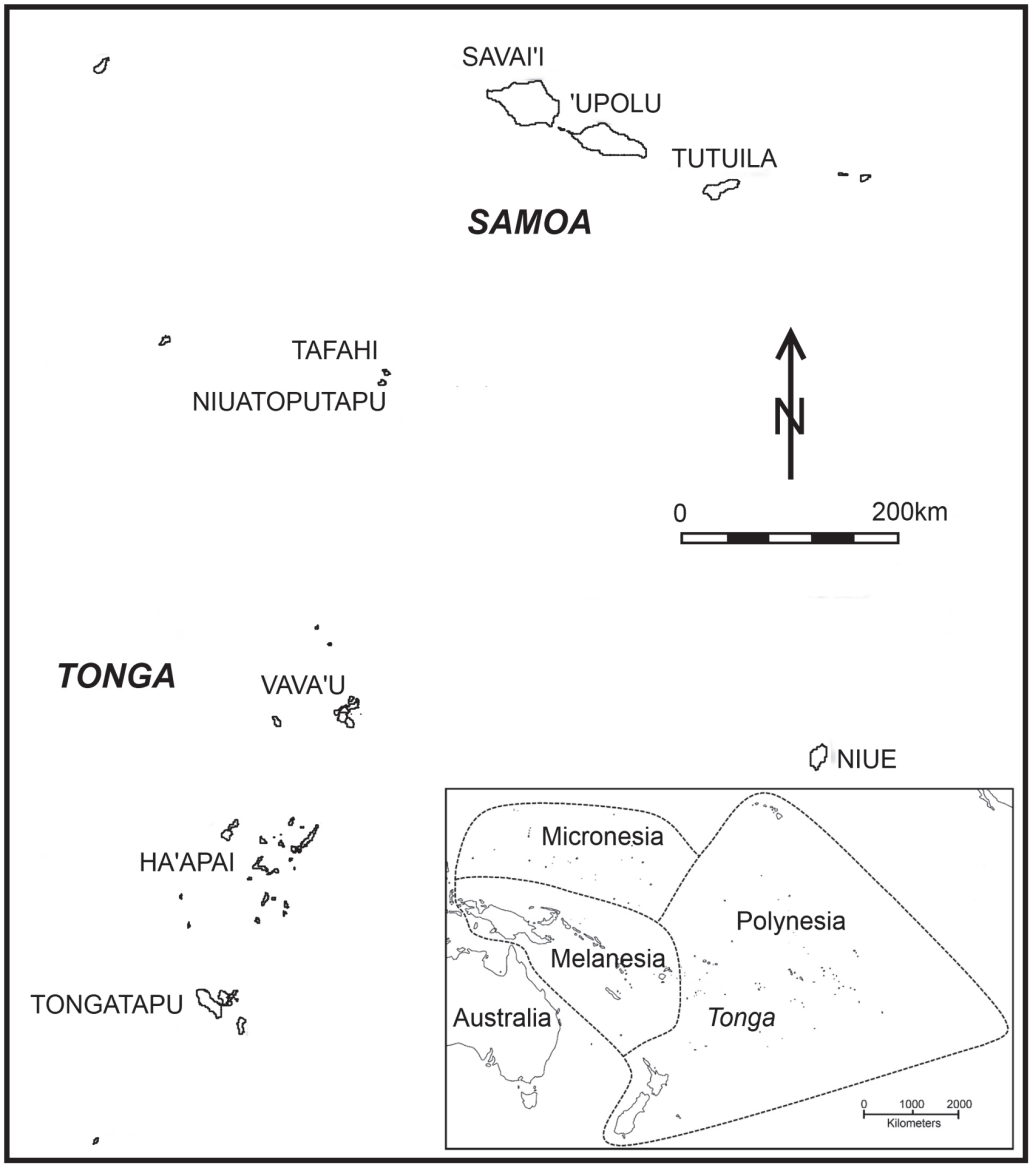
Map of Tonga illustrating island groups examined in text.

The Tongan archipelago consists of more than 160 islands aligned along an 800 km southwest to northeast axis (Fig. 1). From south to north these islands traditionally are grouped into Tongatapu, Ha‘apai and Vavaʻu, with extreme outliers on either end. Archaeology in Tonga has a long history of study [15] with a principal focus being first settlement by “Lapita peoples” [16], [17]. Lapita sites are clearly demarcated by distinctive types of decorated ceramics and other markers. Research questions vary but most reflect in some way on the development of ancestral Polynesian society out of this founding population. Kirch and Green [10] associate the transition from an initial Lapita phase to the Polynesian Plainware phase (loss of decorated ceramics) with nascent development of ancestral Polynesia and the emergence of a Polynesian cultural template. This template in developed form is transferred later into East Polynesia with renewed exploration 1000–1100 cal BP [5]. The substantial degree of cultural homogeneity among widely dispersed Polynesian peoples, as witnessed by Europeans in the late 18^th^ century, is so accounted.

Early research in Tonga demonstrated widespread and rapid colonization throughout the archipelago [18]. Burley initiated his study of Lapita settlement in Ha‘apai in 1991, with later focus shifting to Tongatapu and Vavaʻu. Field studies involve excavations at 12 locales, 11 with Lapita phase ceramics overlain by a Polynesian Plainware phase occupation. Continuity of occupation extends beyond the Polynesian Plainware phase at many sites, and several are the ancestral basis for a modern village. Burley’s excavation methods, recording, and sample collection strategies were consistently applied from the outset. Relative to radiocarbon chronology, shell dates intentionally were excluded given uncertainties of marine reservoir corrections, hardwater influence and the absence of species specific corrections in this region which further complicate the Bayesian analysis [14], [19]. Focus was on the collection of wood charcoals with secure stratigraphic and archaeological association. Samples were measured by accelerator mass spectrometry at Lawrence Livermore National Laboratory (CAMS) in California or, more recently, at the University of Waikato Dating Laboratory (WK) in New Zealand. Notwithstanding currently recognized problems for in-built age, these dates appeared to have a coherent logic in their cumulative representation [20], as outlined in earlier papers on Ha‘apai [11], Tongatapu [12] and Vavaʻu [13].

The AMS radiocarbon dating strategy for Tonga was developed in the early 1990s and the future maelstrom of discontent with unidentified wood charcoal dates was not anticipated by Burley. The possibility of old wood effect was noted, and a small number of outlier dates are explained as a probable consequence. Only one sample from the 12 site excavations was identified as a short-lived sample, this an easily recognized nutshell. In hindsight, failure to identify wood charcoals is a significant problem. Similar oversight in Hawaiian archaeology lead Reith et al. [6] to describe it as a complete “waste of resources that only serves to retard progress in refining the settlement chronology.” Fortunately for Tonga, as stated previously, alternative U/Th dating of coral artifacts provide a high precision age for first settlement at Nukuleka. The earliest date, 2838 ± 8 cal BP (U/Th 11–36) from beach sands immediately below the site, is supported by the short-lived nutshell radiocarbon date (WK 23710), the two occurring in the same stratigraphic context [9].

## Samples

We accumulated new data in three ways to redevelop the Lapita/Polynesian Plainware phase chronology for Tonga. First, 32 of the unidentified wood charcoal dates acquired by Burley had remnant samples retained as an archive. These were re-examined with 11 identified subsequently as coming from short-lived material, all but one being coconut endocarp (S1 Text). We additionally incorporate two coconut endocarp radiocarbon dates recently published by Petchey and Clark [14] bringing the total number of short-lived sample dates to 14. Second, we include five AMS radiocarbon dates on extinct terrestrial iguana bone from a Lapita-age deposit in Haʻapai published by Steadman et al. [21]. While these dates have high stratigraphic integrity and potential for dating first settlement in Haʻapai, in the absence of appropriate bone quality control indicators we cannot rule out potential error due to contamination. We include the iguana bone dates in our analysis, but not as short-lived samples, as justified in Supporting Information (S1 Text). And third, we integrate 14 high precision U/Th dates on coral abraders into the group. Eight of these from the founder settlement of Nukuleka are previously published [9]; the additional six come from five other Lapita phase sites in Tongatapu, Haʻapai and Vavaʻu. The total cumulative sample of dates for analysis is 65; 28 are now high precision U/Th dates or short-lived sample radiocarbon measurements. Table 1 provides cultural phase and island group tabulation for the collection. Additional details are given as Supporting Information (S1 Text).

**Table 1:** Distribution of radiocarbon and U/Th dates by temporal phase and island group.

Phase	Group	Sites	^14^C SL	^14^C Bone	^14^C Char	U/Th	Total
**Lapita**	Tongatapu	2	1		8	9	18
	Haʻapai	5	5	5	8	4	21
	Vavaʻu	4	1		5	1	7
**Plainware**	Tongatapu	2	2		1	0	3
	Haʻapai	6	5		9	0	14
	Vavaʻu	1	0		2	0	2
**Total**			14	5	32	14	65

Abbreviations are short-lived sample dates (^14^C SL), Iguana bone dates (^14^C Bone), unidentified wood charcoal dates (^14^C Char), Uranium Thorium dates on coral file abraders (U/Th).

## Methods and Results

Recent analyses of radiocarbon chronologies in Oceania, whether at the site, island group or regional meta-analysis scale, emphasize and apply Bayesian analysis as a means to identify outlier dates, refine understanding of depositional events or develop chronological models for archaeological sequences [7], [22], [23]. OxCal software for radiocarbon date calibration and analyses considerably facilitates this scholarship. OxCal enables a variety of statistical techniques to be employed in the construction of Bayesian models where prior archaeological information may be integrated; for instance, the sequential or otherwise ordering of stratified archaeological layers [24], [25]. It allows us to re-analyze the Tongan radiocarbon data set for Lapita and Polynesian Plainware phases within the three island groups of Tongatapu, Ha‘apai and Vavaʻu. It also allows us to do this incorporating the more precise U/Th date samples (S1, S2 and S3 Tables). Here we use OxCal 4.2.3 with radiocarbon dates recalibrated employing SHCal13 [26]. Our approach to Bayesian analysis is conservative, where we use the standard boundary command rather than tau or trapezoidal boundaries. The standard boundary command emphasizes abrupt change between the phases, a circumstance we believe is indeed reflected in the Tongan archaeological record.

Prior to Bayesian analysis, we emphasize our continued acceptance of the U/Th date of 2838 ± 8 cal BP (2σ)(U/Th 11–36) from the Nukuleka site on Tongatapu as the expected age for first Lapita landfall in Tonga. We justify this on the basis of three observations. First, Nukuleka is the only site in Tonga with western Lapita ceramics, a characteristic hypothesized for the founding colony. A suite of those ceramics also incorporates temper sands and pastes that are exotic to Tonga (8). Second, the dated *Acropora* coral abrader (U/Th 11–36) was acquired from Stratum IV, the beach sand on which the midden deposit developed. A coral file abrader U/Th date of 2798 ± 8 cal BP (U/Th 11–33)(2σ) from the beach sand/midden transition (Stratum III/IV) appropriately marks the beginning of midden accumulation [9]. And third, the U/Th range of 2830–2846 cal BP (2σ) centrally positions within the calibrated range (68.2%) for the short-lived nutshell radiocarbon date (WK 23710) from the same sub-midden context [9].

### Bayesian Overlap Model for Lapita Phase Settlement in Tonga

With Nukuleka as the founding colony, and no evidence for a second wave of immigration elsewhere in Tonga, we hypothesize a temporal lag for population infilling and growth on the island of Tongatapu prior to Lapita migration into central and northern island groups. To test this hypothesis, gain insight into the extent of this lag, and to develop a precise chronology for Lapita expansion and its duration in each island group, we applied an Oxcal Bayesian overlap model for 46 Lapita-associated dates. Grouped dates for Tongatapu, Ha‘apai and Vavaʻu are categorized as phases, but the overlap model treats each independently without assumption of temporal sequencing or contiguity [24]. The overlap model, thus, does not interject expectations incorporated within the hypothesis. The OxCal Bayesian software provides an agreement index to test the robustness of the model, as well as individual samples. An index threshold of 60% is recommended although, as Bronk Ramsay [27] cautions, “1 in 20 samples are likely to fall below this level” and rejection should be based on logical consideration of other criteria. Our initial run of the Lapita model found three radiocarbon dates with an index threshold less than 60%, two (CAMS 41524, CAMS 119700) substantially so (S1 Text). The latter are clear outliers and, we believe, they are a result of in-built age in the samples. The third date (WK 23708) is only marginally below acceptance (A = 57%), it has good stratigraphic context, and we see no reason for out right rejection. The initial two outliers were removed in our subsequent run of the model resulting in a very high overall agreement index of 247% for the Lapita overlap model. Even more notable, and with exception of the single date just noted, each of the modeled dates has an agreement index of 94% or higher.

**Fig 2:**
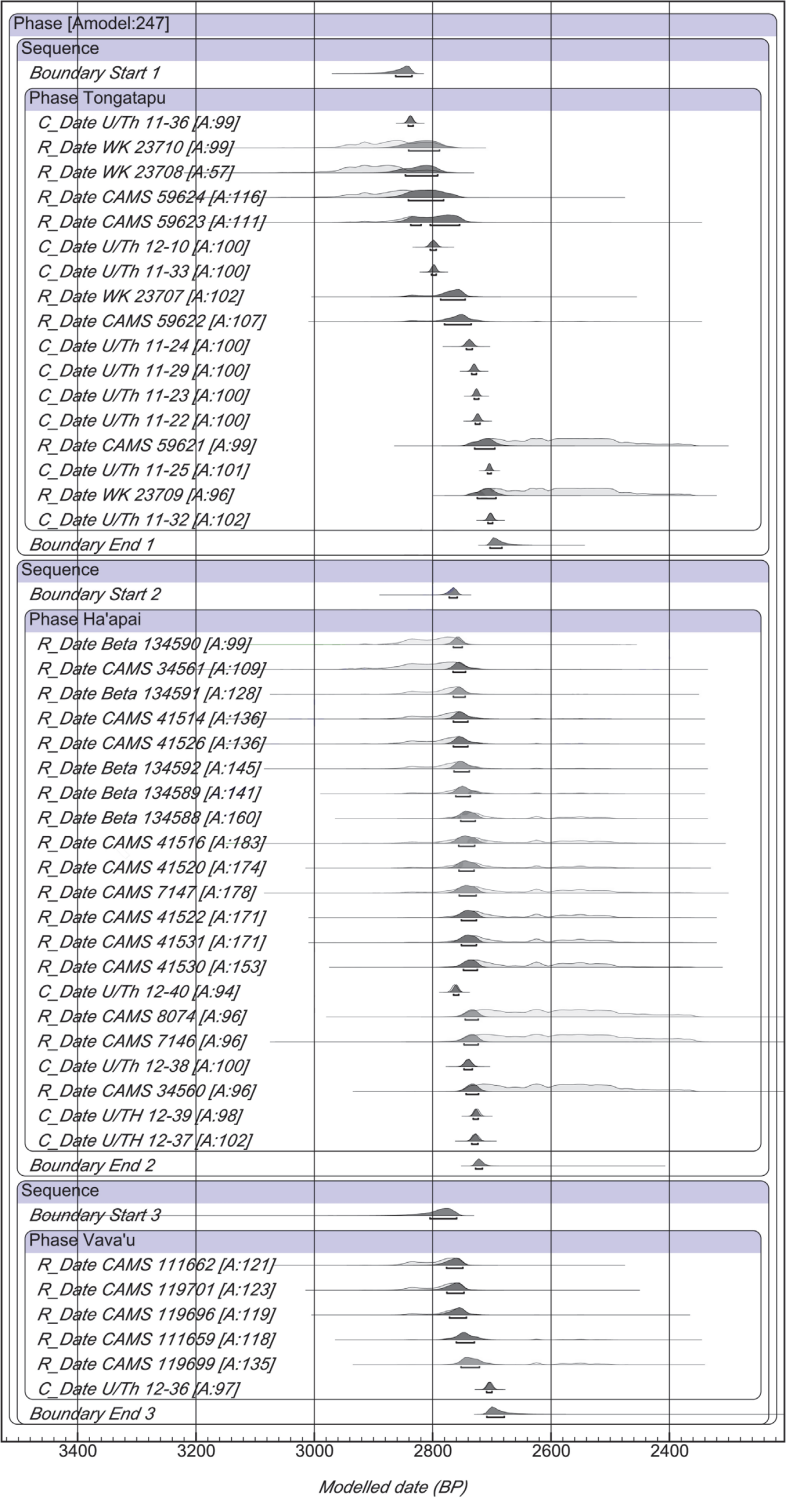
Bayesian overlap model of Tongan Lapita dates. Radiocarbon dates are calibrated using SHCal13 atmospheric curve [26] while the plot is a generated output of OxCal Version 4.2.3. Calibrated age ranges and the model are given at 68.2% probability. S1 Table provides detailed data for individual dates and the model.

The plot of the Lapita overlap model with modeled date ranges at 68.2% probability is provided in Fig. 2 with data given in Supporting Information (S1 Table). We justify 68.2% probability ranges based on the earliest U/Th date at Nukuleka and its central position within the 68.2% range for the nutshell date (WK 23710)[9]. Moreover, the overlap model start date for first settlement on the island of Tongatapu is estimated to be 2863–2835 cal BP. The U/Th date we associate with first establishment of the Nukuleka site is 2846–2830 cal BP (2σ). These ranges are all but identical. For Tongatapu, the end date is 2703–2683 cal BP at 68.2% probability with the duration of the Lapita phase being 129–158 years. We qualify this end date and duration estimate for Lapita in Tongatapu with acknowledgement of there being but two sites represented in our sample. There is a possibility that Lapita ceramic decoration persisted at other sites beyond this range.

The start date for Ha‘apai first settlement is 2772–2759 cal BP at 68.2% probability. Comparing the Ha‘apai and Tongatapu start intervals, we estimate a 70–90 year temporal lag before Lapita colonization of the central island group began. This is approximately four generations after landfall at Nukuleka, a generation length arbitrarily defined as 20 years. Unlike Tongatapu, the Ha‘apai sample of Lapita age dates represents five sites from five different islands. Based on the intensity of survey in these islands, as well as small island size, it is unlikely that a large number of other Lapita-age sites will be found. The modeled Bayesian output dates, therefore, are taken to be representative. The end date for Lapita in Haʻapai is 2728–2716 cal BP (68.2%). The interval between first settlement and the loss of decorated Lapita ceramics in Haʻapai is startling short, between 32 and 49 years in duration.

The islands of Vavaʻu are 130 km north of the largest concentration of Lapita sites in Haʻapai and 300 km north of Tongatapu. If Lapita settlement of Vavaʻu resulted from a progressive movement northward, then additional lag in settlement chronology might be expected. Extensive and multiyear archaeological survey in Vavaʻu recorded five Lapita age sites; excavations were undertaken at four of these [28]. Appropriate charcoal samples with *in situ* context for radiocarbon dating of Vavaʻu sites are rare. We have, therefore, only a limited series of six radiocarbon dates for the Lapita era of Vavaʻu as well as a single U/Th date. One of the Vavaʻu dates was identified and removed as an outlier by the overlap model. The remainder provides a start date of 2805–2760 cal BP, an end date of 2709–2680 cal BP and a Lapita phase duration in Vavaʻu of 51–82 years at 68.2% probability. Expectations for a lag in settlement progression are not met, since the Ha’apai and Vava’u start dates are statistically identical, overlapping at 68.2% probability. We note, however, that the limited numbers of samples for Vava’u, coupled with their position on the radiocarbon calibration curve, increases the density and spread of the start and end posterior probability distributions. The single U/Th date of 2712–2694 cal BP (2σ) (U/Th 12–36) and the short-lived species radiocarbon date of 2752–2721 cal BP (68.2%)(CAMS 119699) alternatively support a more recent settlement start. We continue to hypothesize that first settlement in Vavaʻu will be slightly later than Haʻapai.

### Bayesian Overlap Model for Polynesian Plainware Phase Settlement in Tonga

The transition to the Polynesian Plainware phase in Tonga is exclusively defined by the loss of decoration on ceramic vessels. This is an arbitrary marker since population/cultural continuity is without question, and there are only a few other transitions in related artifact assemblages or cultural features, at least initially. Kirch and Green [10] nevertheless give this transition significantly greater import for Polynesian culture history. To them, the loss of decorated ceramics signals a beginning for the development of ancestral Polynesian society, a Polynesian cultural template and, ultimately, the Proto-Polynesian linguistic substage. As with Lapita, we employ a Bayesian overlap model to estimate start and end dates in each of the island groups. We assume start dates for this phase will in general equate with the previously given Lapita end dates.

**Fig 3:**
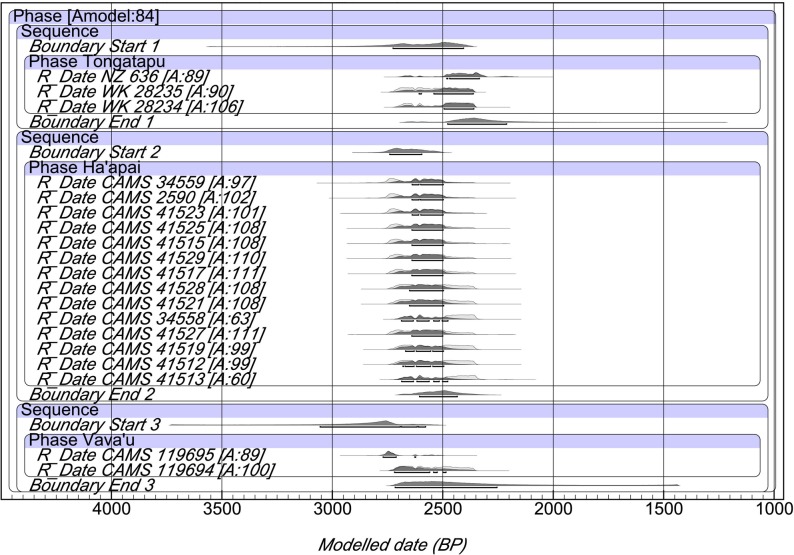
Bayesian overlap model of Tongan Polynesian Plainware dates. Radiocarbon dates are calibrated at 68.2% using SHCal13 atmospheric curve [26] while the plot is a generated output of OxCal Version 4.2.3. S2 Table provides detailed data for individual dates and the model.

The modeled Polynesian Plainware dates are plotted in Fig. 3 at 68.2% probability with data given in supporting information (S2 Table). All radiocarbon dates in the overlap model have agreement indices of 60% or higher with the overall model being 84%. Two observations are immediately apparent. First, there are only three Polynesian Plainware radiocarbon dates for Tongatapu and two for Vavaʻu. This results in greatly skewed posterior probabilities for the start and end archaeological phase ranges, rendering the model inadequate for these island groups. Second, all of the Polynesian Plainware phase dates in Tonga fall within a segment of the radiocarbon calibration curve that is substantially flattened. This is referred to as the “Early Iron Age” or “Hallstatt Plateau” [29]. When radiocarbon ages between 2650 and 2350 BP are calibrated, they typically incorporate the same 250–300 year time range. For Haʻapai, the modeled dates literally are homogeneous, with a start date of 2740–2595 cal BP and an end date of 2605–2435 cal BP at 68.2% probability. The earliest potential start date for Polynesian Plainware in Haʻapai (2740 cal BP) nevertheless corresponds closely with the end date range for Lapita in these islands (2728–2716 cal BP) as expected.

### Bayesian Contiguous Model for Short-lived Chronology in Haʻapai

The series of Haʻapai dates for Lapita and Polynesian Plainware phases incorporates ten short-lived radiocarbon samples and four U/Th dates on *Acropora* coral files. We analyze these dates within a Bayesian contiguous model available from OxCal to evaluate the results of the overlap model for Haʻapai, and to identify any impacts the unidentified wood charcoal samples and bone dates may have had. The contiguous application is a multiphase model that assumes temporally progressive transitions between each phase hence the phases are contiguous without intervening gaps [24]. This is indeed the case for the Lapita and Polynesian Plainware archaeological phases in Haʻapai and elsewhere in Tonga. The contiguous model also helps to refine insight into Lapita settlement in Haʻapai, its duration, and the transition date for the Polynesian Plainware phase.

**Fig 4:**
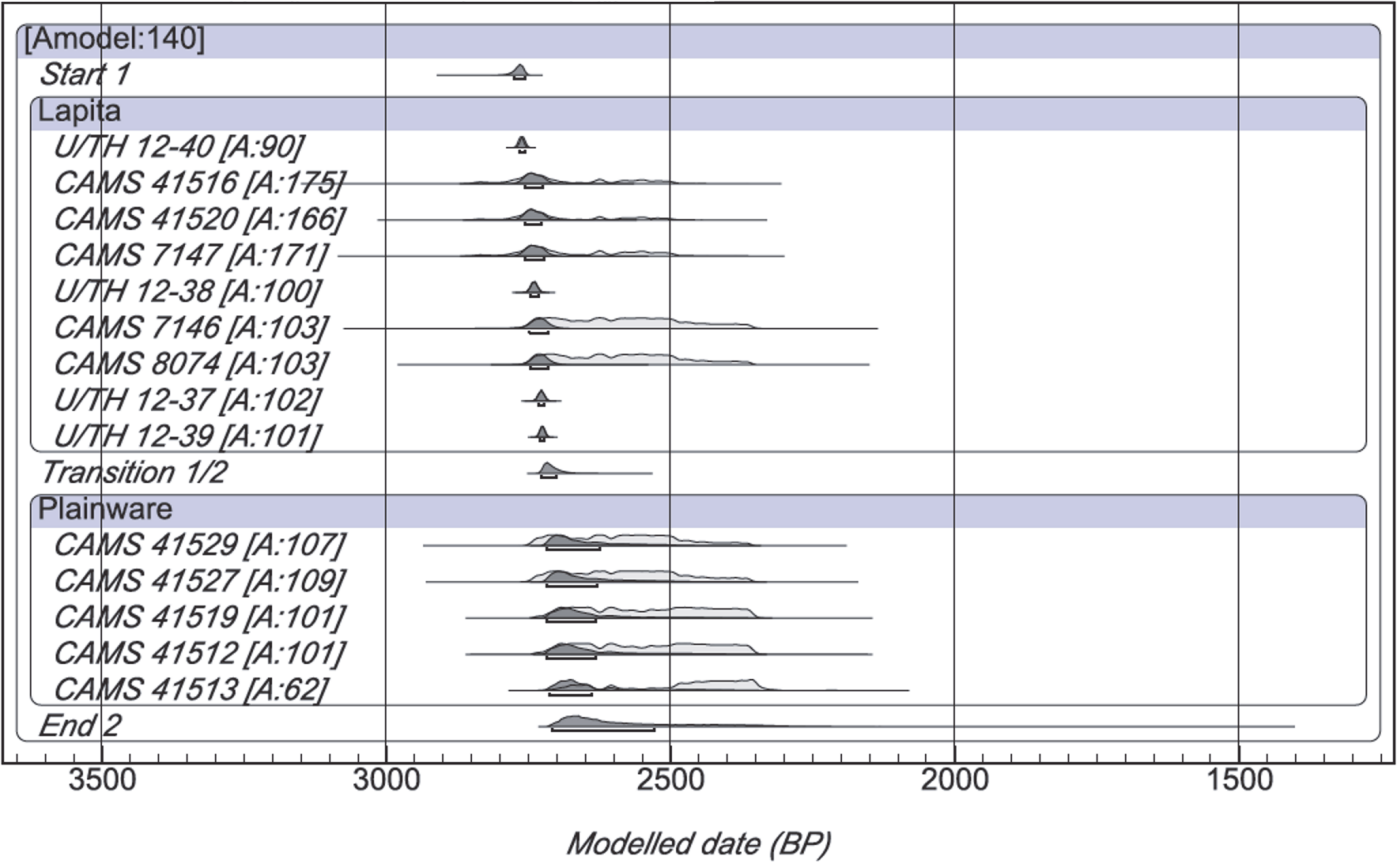
Bayesian contiguous model for short-lived sample and U/Th dates from Haʻapai. Radiocarbon dates are calibrated at 68.2% using SHCal13 atmospheric curve [26] while the plot is a generated output of OxCal Version 4.2.3. S3 Table provides detailed data for individual dates and the model.

The Haʻapai contiguous model has an overall agreement index of 140% and none of the individual dates fall below the 60% threshold (Fig. 4, S3 Table). The start date at 68.2% probability for Lapita in the contiguous model is all but identical to that produced in the Lapita overlap model for Haʻapai, being 2776–2756 cal BP (contiguous) compared to 2772–2759 cal BP (overlap). Unidentified wood charcoal dates and iguana bone dates in the overlap model appear to have little to no influence on modeled outputs. Rather than an end date for the phase as in the overlap model, the contiguous model provides a transition date to the sequent phase. In this case the transition from Lapita to Polynesian Plainware at 68.2% probability occurs between 2726 and 2701 cal BP in Haʻapai with 31–53 years duration for Lapita phase settlement as defined by decorated ceramic wares. The duration of the Lapita phase in Haʻapai is exceedingly short, defined here as a two and a half generation long event at most.

The end of the Polynesian Plainware phase in Tonga is demarcated by the loss of ceramic production altogether. Our modeling of Polynesian Plainware phase dates in the overlap model for Tonga or the contiguous model for Haʻapai leaves open the question of a boundary end. On the one hand, we have grouped dates in Haʻapai suggesting a temporal extent of 300 or so years. On the other, the overlap model end dates for Tongatapu and Vavaʻu potentially anticipate a considerably longer sequence. Burley [15] suggested the latter situation previously, with a possibility that Polynesian Plainware ceramics continued to be produced until 1550 cal BP. Kirch [18] extends this even further for the far northern island of Niuatoputapu, suggesting pottery loss was not a simultaneous occurrence throughout the archipelago.

## Discussion

**Fig 5:**
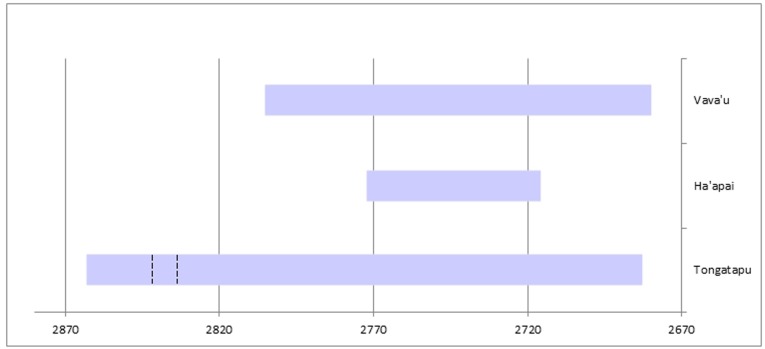
Modeled timelines for Lapita phase settlement in Tonga. Boundary ranges are the earliest and lates possible start/end date for Lapita for each group in the overlap model at 68.2% probability. The ranges are 2863–2683 cal BP for Tongatapu, 2772–2716 cal BP for Haʻapai and 2805–2680 cal BP for Vavaʻu. The dashed segment plots U/Th 11–36 (2838 ± 8 cal BP, 95.4%), a date relating to the founder event for human settlement [9].

Oceanic archaeology is in the midst of chronological reassessment, where chronometric hygiene protocols for sample identifications and use of short-lived samples is pre-eminent. A substantial shortening of East Polynesian archaeological sequences has been a consequence but these protocols raise questions for other chronologies where radiocarbon dates do not meet the standard. The radiocarbon sequence for Tonga is among the latter. As Tonga is the first island group settled in Polynesia, this becomes problematic for answering “when questions” associated with Polynesian origins and ancestral Polynesian society. To reestablish the Tongan chronology, and to refine its precision for interpretation, we add a series of short-lived sample dates for wood charcoal and we present a series of high precision U/Th dates for Lapita/Polynesian Plainware sites across the archipelago. Bayesian analysis of these data incorporated with unidentified wood charcoal radiocarbon dates and iguana bone dates provides new chronological insight for each of Tonga’s three island groups. A graphic chronology of the resulting timeline is provided as Fig. 5. We summarize our conclusions and implications as follows.

1. First Lapita landfall in Polynesia occurred at the Nukuleka site on the southern island of Tongatapu as documented in ceramic style and origins for the earliest assemblage. The U/Th age interval of 2846–2830 cal BP (2σ)(U/Th 11–36) provides a measure of 16 years within which this landfall seemingly occurred. Our Bayesian overlap model of 17 Lapita dates for the island of Tongatapu provides a closely comparable interval of 2863–2835 cal BP (68.2%) for first Lapita settlement. This illustrates not just the veracity of the Nukuleka U/Th date, but reflects upon the degree of accuracy for the overlap model itself.
2. Between 2772 and 2759 cal BP (68.2%), 70–90 years after first landfall at Nukuleka, Lapita peoples moved northward into the principal islands of Haʻapai. No less than six of these islands were colonized immediately thereafter. In addition to this movement, contemporaneous expansion was occurring around the Tongatapu lagoon as well as across the Nukuleka peninsula [21]. We can only surmise that population growth rates on Tongatapu in the four or five generations following initial settlement must have been incredibly high or that additional in-migration was taking place. There is no direct evidence to argue for the latter as yet.
3. Lapita dates for Vavaʻu provide a 68.2% probability range of 2805–2760 cal BP for first settlement, this overlapping with and being statistically indistinct with the modeled first settlement date for Haʻapai. The number of dates for Vavaʻu is limited however. Based on a single short-lived sample radiocarbon date and a single U/Th date for Lapita in Vavaʻu, we continue to hypothesize Vava’u colonization as being slightly delayed from Haʻapai. We extend this interpretation to incorporate settlement in the far northern outlier of Niuatoputapu. We have no acceptable Lapita-age radiocarbon or U/Th dates for Niuatoputapu, but Burley et al. [30] document Niuatoputapu volcanic glass in Lapita occupations of all three of the principal island groups. This illustrates simultaneous Lapita settlement for Niuatoputapu as well as interisland voyaging and communication along the length of the archipelago at this early time.
4. The Lapita phase in Tonga is defined exclusively by the presence of decorated ceramics. In its earliest manifestations at Nukuleka, these designs are complex, densely applied and have motifs typical of Lapita ceramics in central/western Melanesia [21]. This complexity is transformed at other Lapita sites in Tonga where motifs are simplified, motif application is open and decorative applications are less well executed. The process ends with the disappearance of decoration, a point at which the Lapita phase ends and the Polynesian Plainware phase begins. The duration of the Lapita phase in each of the three island groups provides a temporal measure to gauge the rapidity with which simplification and loss took place. Based on the Bayesian overlap model at 68.2% probability, this span is calculated to be 129 to 158 years on Tongatapu, 32 to 49 years in Haʻapai, and 51 to 82 years in Vavaʻu. When viewing this in terms of potter generations of 20 year intervals, the chronology is of remarkably short duration. For Tongatapu it is 6.5 to 8 generations, for Haʻapai it is 1.5 to 2.5 generations, and for Vavaʻu it is 2 to 4 generations or quite possibly less. The Haʻapai number is exceptional; it means that first and second generation potters on these islands produced the full Lapita ceramic assemblage as it has been excavated. Third generation potters stopped making decorated pottery altogether.
5. The 68.2% probability ranges for Lapita end dates for each of the island groups in the overlap model provides comparable measures for ceramic transition across the archipelago. This transition similarly marks the onset of ancestral Polynesian society in the view of Kirch and Green [10]. The transition occurs first in Haʻapai at 2728–2716 cal BP and then slightly later at 2703–2683 cal BP for Tongatapu and 2709–2680 cal BP for Vavaʻu. The differences are small, and the loss of decorated pottery is taken to be roughly simultaneous throughout Tonga. When the third generation Haʻapai potters stopped making decorated pots, whatever their reasons may have been, they were part of a much larger pattern of change.
6. Finally, we believe this paper provides a solution to sites where strict chronometric hygiene protocols for radiocarbon dating would have removed all charcoal-based radiocarbon dates where wood species or sample materials remain unidentified. In the Tongan case, the radiocarbon chronology would have been eliminated if this were the case. In re-examining the archived record of dated samples, 11 of 32 potentially identifiable charcoals were found to be of short-lived material, predominantly of coconut endocarp. We also note that the remaining 21 samples could possibly be short-lived; they could not be identified as to species because of sample size or because of a lack of reference material. Second, the use of Bayesian models to identify outlier dates is well developed in the OxCal open source software and discussed extensively by Bronk Ramsay [27]. In our case, this has led to the rejection of only two radiocarbon dates where wood identifications have not been made. Thus, of the 46 accepted radiocarbon dates we initially started with, 44 (95.7%) are robust when integrated into a Bayesian model with high precision U/Th dates. Similarly, the five dates on extinct iguana bone from Ha'apai also fit well within the Bayesian overlap model, despite the absence of appropriate quality control indicators for bone dates. In this example, the use of these dates, in concert with Bayesian modeling also incorporating short-lived sample dates and high-precision U/Th dates, has added substantially to our understanding of the colonization and subsequent expansion of peoples within the ancestral Polynesian homeland.

## Supporting Information

S1 Table Bayesian overlap model for Lapita phase dates in the Kingdom of Tonga. Radiocarbon dates are calibrated at 68.2% using SHCal13 atmospheric curve [16]. The overall model agreement is 247%. Abbreviations are Bayesian model range (Model BP), agreement indice (Agree), published reference for date (Ref), short-lived charcoal (SL Char), unidentified wood char (char) and here-to-fore unpublished date (UP). Modeled ranges are plotted in Fig. **2**.
(DOCX)

S2 Table Bayesian overlap model for Polynesian Plainware phase dates in the Kingdom of Tonga. Radiocarbon dates are calibrated at 68.2% probability using SHCal13 atmospheric curve [16]. The overall model agreement is 84%. Abbreviations are Bayesian model range (Model BP), agreement indice (Agree), published reference for date (Ref), short-lived charcoal (SL Char), unidentified wood char (char) and here-to-fore unpublished date (UP). Modelled ranges are plotted in Fig. 3.
(DOCX)

S3 Table Bayesian contiguous model for short-lived sample and U/Th dates in Ha’apai. Radiocarbon dates are calibrated at 68.2% using SHCal13 atmospheric curve [16]. The overall model agreement is 140%. Abbreviations are Bayesian model range (Model BP), agreement indice (Agree), published reference for date (Ref), short-lived charcoal (SL Char), unidentified wood char (char) and here-to-fore unpublished date (UP). Modeled ranges are plotted in Fig. 4.
(DOCX)

S1 Text Description of dated samples used within the analysis. (DOCX)
