# Supplementary material for: Bayesian Modeling and Chronological Precision for Polynesian Settlement of Tonga
Source: PLoS One. 2015 Mar 23;10(3):e0120795. doi: 10.1371/journal.pone.0120795 (PMC4370570; doi:10.1371/journal.pone.0120795)
Supplement: S1 Table — Radiocarbon dates are calibrated at 68.2% using SHCal13 atmospheric curve [16]. The overall model agreement is 247%. Abbreviations are Bayesian model range (Model BP), agreement indice (Agree), published reference for date (Ref), short-lived charcoal (SL Char), unidentified wood char (char) and here-to-fore unpublished date (UP). Modeled ranges are plotted in Fig. 2 . (DOCX) [file pone.0120795.s001.docx]

|  | **Site Name** | **^14^C Date** | **Material** | **Cal BP** | **Model BP** | **Agree** | **Ref** |
| --- | --- | --- | --- | --- | --- | --- | --- |
|  |  |  |  |  |  |  |  |
| **TONGATAPU** | |  |  |  |  |  |  |
|  |  |  |  |  |  |  |  |
| U/Th 11-36 | Nukuleka |  | coral | 2842-2834 | 2841-2833 | 99 | [13] |
| WK 23710 | Nukuleka | 2811±35 | SL char | 2923-2793 | 2841-2789 | 99 | [4] |
| WK 23708 | Nukuleka | 2836±32 | char | 2950-2852 | 2847-2792 | 57 | [4] |
| CAMS 59624 | Nukuleka | 2790±50 | char | 2919-2776 | 2841-2782 | 116 | [2] |
| CAMS 59623 | Ha'ateiho | 2730±50 | char | 2844-2754 | 2837-2755 | 111 | [2] |
| U/Th 12-10 | Ha'ateiho |  | coral | 2806-2792 | 2804-2794 | 100 | UP |
| U/Th 11-33 | Nukuleka |  | coral | 2802-2794 | 2802-2794 | 100 | [13] |
| WK 23707 | Nukuleka | 2696±32 | char | 2788-2745 | 2787-2745 | 102 | [4] |
| CAMS 59622 | Ha'ateiho | 2670±40 | char | 2783-2730 | 2780-2735 | 107 | [2] |
| U/Th 11-24 | Nukuleka |  | coral | 2745-2731 | 2743-2733 | 100 | [13] |
| U/Th 11-29 | Nukuleka |  | coral | 2734-2726 | 2734-2726 | 100 | [13] |
| U/Th 11-23 | Nukuleka |  | coral | 2730-2722 | 2730-2722 | 100 | [13] |
| U/Th 11-22 | Nukuleka |  | coral | 2728-2720 | 2728-2720 | 100 | [13] |
| CAMS 59621 | Ha'ateiho | 2540±40 | char | 2720-2492 | 2730-2695 | 99 | [2] |
| U/Th 11-25 | Nukuleka |  | char | 2707-2701 | 2707-2701 | 101 | [13] |
| WK 23709 | Nukuleka | 2536±32 | char | 2717-2493 | 2725-2701 | 96 | [4] |
| U/Th 11-32 | Nukuleka |  | coral | 2706-2698 | 2707-2699 | 102 | [13] |
|  |  |  |  |  |  |  |  |
| **Lapita Start** |  |  |  |  | 2863-2835 |  |  |
| **Lapita End** |  |  |  |  | 2703-2683 |  |  |
| **Span (years)** |  |  |  |  | 129-158 |  |  |
|  |  |  |  |  |  |  |  |
| **HA'APAI** |  |  |  |  |  |  |  |
|  |  |  |  |  |  |  |  |
| Beta 134590 | Tongoleleka | 2730±40 | iguana | 2843-2755 | 2765-2750 | 98 | [6] |
| CAMS 34561 | Tongoleleka | 2720±60 | char | 2844-2750 | 2766-2745 | 109 | [1] |
| Beta 134591 | Tongoleleka | 2700±40 | iguana | 2840-2744 | 2765-2745 | 128 | [6] |
| CAMS 41514 | Tongoleleka | 2690±50 | char | 2842-2738 | 2765-2740 | 136 | [1] |
| CAMS 41526 | Vaipuna | 2690±50 | char | 2842-2738 | 2765-2740 | 136 | [1] |
| Beta 134592 | Tongoleleka | 2680±50 | iguana | 2841-2725 | 2764-2738 | 145 | [6] |
| Beta 134589 | Tongoleleka | 2660±40 | iguana | 2777-2724 | 2761-2736 | 141 | [6] |
| Beta 134588 | Tongoleleka | 2630±40 | iguana | 2766-2543 | 2753-2728 | 160 | [6] |
| CAMS 41516 | Pukotala | 2640±60 | SL char | 2784-2514 | 2756-2730 | 183 | [6] |
| CAMS 41520 | Mele Havea | 2640±50 | SL char | 2782-2540 | 2756-2730 | 174 | [1] |
| CAMS 7147 | Pukotala | 2630±60 | SL char | 2775-2507 | 2755-2727 | 178 | [1] |
| CAMS 41522 | Mele Havea | 2620±50 | char | 2761-2519 | 2752-2726 | 171 | [1] |
| CAMS 41531 | Vaipuna | 2620±50 | char | 2761-2519 | 2752-2726 | 171 | [1] |
| CAMS 41530 | Faleloa | 2600±50 | char | 2752-2505 | 2748-2725 | 153 | [1] |
| U/Th 12-40 | Vaipuna |  | coral | 2768-2758 | 2765-2756 | 94 | UP |
| CAMS 8074 | Faleloa | 2560±60 | SL char | 2741-2493 | 2745-2723 | 96 | [1] |
| CAMS 7146 | Faleloa | 2560±60 | SL char | 2742-2491 | 2747-2723 | 96 | [1] |
| U/Th 12-38 | Tongoleleka |  | coral | 2748-2733 | 2747-2733 | 100 | UP |
| CAMS 34560 | Tongoleleka | 2560±50 | char | 2739-2495 | 2744-2723 | 96 | [1] |
| U/TH 12-39 | Tongoleleka |  | coral | 2730-2720 | 2731-2723 | 98 | UP |
| U/TH 12-37 | Faleloa |  | coral | 2734-2720 | 2734-2724 | 102 | UP |
|  |  |  |  |  |  |  |  |
| **Lapita Start** |  |  |  |  | 2772-2759 |  |  |
| **Lapita End** |  |  |  |  | 2728-2716 |  |  |
| **Span (years)** |  |  |  |  | 32-49 |  |  |
| **VAVA'U** |  |  |  |  |  |  |  |
|  |  |  |  |  |  |  |  |
| CAMS 111662 | Vuna | 2715±35 | char | 2841-2749 | 2777-2749 | 121 | [3] |
| CAMS 119701 | Otea | 2705±35 | char | 2840-2746 | 2776-2747 | 123 | [3] |
| CAMS 119696 | Falevai | 2685±35 | char | 2786-2741 | 2772-2743 | 119 | [3] |
| CAMS 111659 | Vuna | 2650±35 | char | 2767-2721 | 2760-2730 | 118 | [3] |
| CAMS 119699 | Ofu | 2625±35 | SL char | 2760-2545 | 2752-2721 | 135 | [3] |
| U/Th 12-36 | Ofu |  | coral | 2708-2698 | 2709-2700 | 97 | UP |
|  |  |  |  |  |  |  |  |
| **Lapita Start** |  |  |  |  | 2805-2760 |  |  |
| **Lapita End** |  |  |  |  | 2709-2680 |  |  |
| **Span** |  |  |  |  | 51-82 yrs |  |  |

S1 Table. Bayesian Overlap Model for Lapita phase dates in the Kingdom of Tonga. Radiocarbon dates are calibrated at 68.2% using SHCal13 atmospheric curve [16]. The overall model agreement is 247%. Abbreviations are Bayesian model range (Model BP), agreement indice (Agree), published reference for date (Ref), short-lived charcoal (SL Char), unidentified wood char (char) and here-to-fore unpublished date (UP). Modeled ranges are plotted in Fig. 2.
