# Supplementary material for: Bayesian Modeling and Chronological Precision for Polynesian Settlement of Tonga
Source: PLoS One. 2015 Mar 23;10(3):e0120795. doi: 10.1371/journal.pone.0120795 (PMC4370570; doi:10.1371/journal.pone.0120795)
Supplement: S2 Table — Radiocarbon dates are calibrated at 68.2% probability using SHCal13 atmospheric curve [16]. The overall model agreement is 84%. Abbreviations are Bayesian model range (Model BP), agreement indice (Agree), published reference for date (Ref), short-lived charcoal (SL Char), unidentified wood char (char) and here-to-fore unpublished date (UP). Modelled ranges are plotted in Fig. 3. (DOCX) [file pone.0120795.s002.docx]

|  | **Site Name** | **^14^C Date** | **Material** | **Cal BP** | **Model BP** | **Agree** | **Ref** |
| --- | --- | --- | --- | --- | --- | --- | --- |
|  |  |  |  |  |  |  |  |
| **TONGATAPU** | |  |  |  |  |  |  |
|  |  |  |  |  |  |  |  |
| NZ 636 | Tufumahina | 2380±51 | char | 2460-2312 | 2482-2335 | 89 | [5] |
| WK 28235 | Talasiu | 2510±31 | SL char | 2702-2465 | 2607-2362 | 90 | [5] |
| WK 28234 | Talasiu | 2473±31 | SL char | 2678-2361 | 2494-2360 | 106 | [5] |
|  |  |  |  |  |  |  |  |
| **Plainware Start** | |  |  |  | 2725-2406 |  |  |
| **Plainware End** |  |  |  |  | 2478-2211 |  |  |
|  |  |  |  |  |  |  |  |
| **HA'APAI** |  |  |  |  |  |  |  |
|  |  |  |  |  |  |  |  |
| CAMS 34559 | Tongolekea | 2600±60 | Char | 2753-2500 | 2639-2498 | 97 | [1] |
| CAMS 2590 | Holopeka | 2590±60 | char | 2750-2498 | 2639-2498 | 102 | [1] |
| CAMS 41523 | Vaipuna | 2580±50 | char | 2745-2500 | 2639-2499 | 101 | [1] |
| CAMS 41525 | Vaipuna | 2560±80 | char | 2739-2495 | 2640-2499 | 108 | [1] |
| CAMS 41515 | Pukotala | 2560±50 | char | 2739-2495 | 2640-2498 | 108 | [1] |
| CAMS 41529 | Faleloa | 2550±50 | SL char | 2731-2493 | 2640-2499 | 110 | [1] |
| CAMS 41517 | Pukotala | 2540±50 | char | 2722-2491 | 2640-2500 | 111 | [1] |
| CAMS 41528 | Holopeka | 2510±50 | char | 2705-2461 | 2650-2497 | 108 | [1] |
| CAMS 41521 | Mele Havea | 2510±50 | char | 2705-2461 | 2651-2496 | 108 | [1] |
| CAMS 34558 | Tongoleleka | 2450±40 | char | 2676-2353 | 2687-2477 | 63 | [1] |
| CAMS 41527 | Holopeka | 2540±50 | SL char | 2722-2491 | 2640-2500 | 111 | [1] |
| CAMS 41519 | Mele Havea | 2490±50 | SL char | 2696-2364 | 2669-2497 | 99 | [1] |
| CAMS 41512 | Tongoleleka | 2490±51 | SL char | 2696-2364 | 2681-2496 | 99 | [1] |
| CAMS 41513 | Tongoleleka | 2430±50 | SL char | 2676-2346 | 2686-2478 | 60 | [1] |
|  |  |  |  |  |  |  |  |
| **Plainware Start** | |  |  |  | 2740-2595 |  |  |
| **Plainware End** |  |  |  |  | 2605-2435 |  |  |
|  |  |  |  |  |  |  |  |
| **VAVA'U** |  |  |  |  |  |  |  |
|  |  |  |  |  |  |  |  |
| CAMS 119695 | Falevai | 2645±35 | unid char | 2765-2719 | 2770-2623 | 89 | [3] |
| CAMS 119694 | Falevai | 2500±35 | unid char | 2700-2433 | 2719-2486 | 100 | [3] |
|  |  |  |  |  |  |  |  |
| **Plainware Start** | |  |  |  | 3055-2578 |  |  |
| **Plainware End** |  |  |  |  | 2716-2255 |  |  |

**S2 Table. Bayesian Overlap Model for Polynesian** **Plainware phase dates in the Kingdom of Tonga.** Radiocarbon dates are calibrated at 68.2% probability using SHCal13 atmospheric curve [16]. The overall model agreement is 84%. Abbreviations are Bayesian model range (Model BP), agreement indice (Agree), published reference for date (Ref), short-lived charcoal (SL Char), unidentified wood char (char) and here-to-fore unpublished date (UP). Modelled ranges are plotted in Fig. 3.
