# Supplementary material for: Bayesian Modeling and Chronological Precision for Polynesian Settlement of Tonga
Source: PLoS One. 2015 Mar 23;10(3):e0120795. doi: 10.1371/journal.pone.0120795 (PMC4370570; doi:10.1371/journal.pone.0120795)
Supplement: S3 Table — Radiocarbon dates are calibrated at 68.2% using SHCal13 atmospheric curve [16]. The overall model agreement is 140%. Abbreviations are Bayesian model range (Model BP), agreement indice (Agree), published reference for date (Ref), short-lived charcoal (SL Char), unidentified wood char (char) and here-to-fore unpublished date (UP). Modeled ranges are plotted in Fig. 4. (DOCX) [file pone.0120795.s003.docx]

|  | **Site Name** | **^14^C Date** | **Material** | **Cal BP** | **Model BP** | **Agree** | **Ref** |
| --- | --- | --- | --- | --- | --- | --- | --- |
| **LAPITA** |  |  |  |  |  |  |  |
|  |  |  |  |  |  |  |  |
| U/Th 12-40 | Vaipuna |  | coral | 2768-2758 | 2765-2755 | 90 | UP |
| CAMS 41516 | Pukotala | 2640±60 | SL char | 2784-2514 | 2756-2725 | 175 | [1] |
| CAMS 41520 | Mele Havea | 2640±50 | SL char | 2782-2540 | 2756-2726 | 166 | [1] |
| CAMS 7147 | Pukotala | 2630±60 | SL char | 2775-2507 | 2755-2723 | 171 | [1] |
| U/Th 12-38 | Tongoleleka |  | coral | 2748-2733 | 2747-2733 | 100 | UP |
| CAMS 7146 | Faleloa | 2560±60 | SL char | 2742-2491 | 2748-2716 | 103 | [1] |
| CAMS 8074 | Faleloa | 2560±60 | SL char | 2741-2493 | 2746-2716 | 103 | [1] |
| U/TH 12-37 | Faleloa |  | coral | 2734-2720 | 2733-2722 | 102 | UP |
| U/TH 12-39 | Tongoleleka |  | coral | 2730-2720 | 2730-2731 | 101 | UP |
|  |  |  |  |  |  |  |  |
| **Lapita Start** |  |  |  |  | 2776-2756 |  |  |
| **Lapita Span (Years)** | |  |  |  | 31-53 |  |  |
| **Lapita/Plainware Transition** | | |  |  | 2726-2701 |  |  |
|  |  |  |  |  |  |  |  |
| **PLAINWARE** | |  |  |  |  |  |  |
|  |  |  |  |  |  |  |  |
| CAMS 41529 | Faleloa | 2550±50 | SL char | 2731-2493 | 2718-2614 | 109 | [1] |
| CAMS 41527 | Holopeka | 2540±50 | SL char | 2722-2491 | 2718-2613 | 109 | [1] |
| CAMS 41519 | Mele Havea | 2490±50 | SL char | 2696-2364 | 2716-2599 | 100 | [1] |
| CAMS 41512 | Tongoleleka | 2490±51 | SL char | 2696-2364 | 2716-2599 | 100 | [1] |
| CAMS 41513 | Tongoleleka | 2430±50 | SL char | 2676-2346 | 2713-2598 | 64 | [1] |

**S3 Table. Bayesian Contiguous Model for short-lived sample and U/Th dates in Ha’apai.** Radiocarbon dates are calibrated at 68.2% using SHCal13 atmospheric curve [16]. The overall model agreement is 140%. Abbreviations are Bayesian model range (Model BP), agreement indice (Agree), published reference for date (Ref), short-lived charcoal (SL Char), unidentified wood char (char) and here-to-fore unpublished date (UP). Modeled ranges are plotted in Fig. 4.
