# Supplementary material for: Bayesian Modeling and Chronological Precision for Polynesian Settlement of Tonga
Source: PLoS One. 2015 Mar 23;10(3):e0120795. doi: 10.1371/journal.pone.0120795 (PMC4370570; doi:10.1371/journal.pone.0120795)
Supplement: S1 Text — (DOCX) [file pone.0120795.s004.docx]

**Supporting Information**

**S1 Text. Description of Dated Samples Used Within the Analysis.**

To redevelop the Lapita/Plainware phase chronology for Tonga, we employ three categories of dates – radiocarbon dates based on charcoal, radiocarbon dates based on terrestrial iguana, and U/Th dates from *Acropora* coral abraders. Each group is discussed briefly to explain the effective sample of dates employed in our analysis. A distributional summary of these dates by phase and island group is provided in Table 1. S1, S2 and S3 Tables provide uncorrected radiocarbon ages as well as calibrated (68.2%) radiocarbon and U/Th dates as they are integrated into overlap or contiguous Bayesian models.

**Radiocarbon Dates on Wood Charcoal**

Burley’s original inventory of radiocarbon dates from Lapita and Polynesian Plainware phase occupations included 48 AMS wood charcoal dates with only one identified as a short-lived sample, this being a nutshell (WK 23710). All of these dates are previously reported [1] – [4]. To this we add three additional charcoal dates from excavations by other researchers, two (WK 28234 , WK 28235) being short-lived coconut endocarp dates for Polynesian Plainware phase deposits on Tongatapu [5]. Five dates have been identified as outliers in previous publications; we continue to omit these outliers from further consideration.

AMS radiocarbon dates previously measured at the Lawrence Livermore National Laboratory were pretreated and combusted at Simon Fraser University with unneeded charcoal archived. Burley re-examined the archive in 2012 to identify samples where charcoal identification might be possible. Thirty-two samples were sent to the Wood Identification Laboratory, International Archaeological Research Institute, Honolulu. Five samples were recognized as including short-lived coconut endocarp charcoals exclusively, one was an unidentified nut, and one other was an amorphous tissue, possibly from a fruit. Four other samples were predominantly coconut endocarp but had small bits of wood charcoal present (CAMS 41520, CAMS 7146, CAMS 7147, CAMS 8074); only one of the wood charcoals could be identified as pandanus. We treat these latter dates as coming from short-lived samples given logical probability that larger fragments of endocarp were selected over small wood bits for dating. With reanalysis and our inclusion of the three additional samples, the charcoal based radiocarbon record for Tonga now incorporates 46 dates from multiple sites across the three island groups. All dates are counted directly by AMS with exception of a single Polynesian Plainware phase date (NZ 636). In the first run of the Bayesian overlap model for Lapita associated dates, two had exceptionally low agreement indices. These dates (CAMS 41524, 2760±50 BP, A=4%; CAMS 119700, 2765±35 BP, A=28%) were removed as outliers probably resulting from inbuilt age. The coconut endocarp, nuts and amorphous tissue dates relate to a single year or growing season having in-built age of no more than one to one and a half years.

**Radiocarbon Dates on Terrestrial Iguana –** ***Brachylophus gibbonsi***

Excavations by Burley in 1995 recovered an abundant collection of extinct/extirpated birds and a large iguana species in the lower Lapita levels of the Tongoleleka site in Ha‘apai. Steadman [6] conducted further tests in 1997 to recover additional fauna. To gauge suddenness of faunal loss, he acquired a series of AMS radiocarbon dates on chicken (*Gallus gallus*)(n=6), extinct megapode (*Megapodus alimentum*)(n=8) and extinct iguana (*Brachylophus gibbonsi*) (n=6). We include in our sample the extinct iguana dates excavated from the Tongoleleka Lapita stratum. The iguana is considered to be exclusively herbivorous, as is the case with contemporary Fijian and Tongan iguana species (*Brachylophus fasciatus, Brachylophus vitiensis*) [7]. This assumption is supported by a mean ^13^C/^12^C isotopic ratio of -21.05 [6]. Chicken and megapode, on the other hand, are known omnivores. Their respective ^13^C/^12^C mean isotopic ratios of -17.2 and – 19.96 could indicate at least a partial intake of marine foods, the chicken more so than the megapode. We omit these dates from consideration, as they potentially require a marine reservoir offset correction. Modern Fijian iguana species have a generation length of between 10 and 15 years [7], albeit they are somewhat smaller than *Brachylophus gibbonsi* [8]. Of the six iguana dates from Tongoleleka, one [6] is much too old for the Polynesian Plainware phase stratum it occurs in. We omit this date as a result. The five AMS radiocarbon dates on iguana are in association with the Lapita occupation at Tongoleleka.

A reviewer for this paper challenged our use of iguana bone dates since we did not have appropriate quality control information to demonstrate minimal contamination. Steadman’s [6] bone dates were processed by Beta analytic who would have used then existing commercial protocols for collagen extraction and measurement. The absence of control data notwithstanding, we see no prior reason to question the accuracy of these dates. The dates are within the range we would expect for first settlement in Ha’apai, the ^13^C/^12^C isotopic ratios are appropriate for a terrestrial species, the dates were not identified as outliers in our initial run of the Bayesian outlier model, and the contiguous model shows little difference in Lapita phase age intervals in Ha’apai (without iguana) relative to the overlap model (with iguana). We also ran the contiguous model twice, one with the iguana dates and the second without. There was no more than a 2-3 year variation in age intervals for the models.

**U/Th Series Dates**

Uranium/thorium dating of corals in Pacific archaeology has been successfully applied in Hawai‘i [9] – [11] and Tahiti [12]. More recently Burley et al. [13] have used this method to date *Acropora* coral abraders from Nukuleka, the founder settlement for Lapita colonization of Tonga. U/Th dates from Nukuleka have a significant advantage over even short-lived wood charcoal dates in that they are direct dates on artifacts, they provide ranges requiring no further calibration, and the standard error is extremely low providing a very high degree of precision. The earliest Lapita settlement at Nukuleka occurs beneath a late prehistoric burial mound. Six coral abraders were dated in vertical sequence through the undisturbed part of the Lapita occupation layer. Seven additional samples from the secondary mound fill, and from a dispersed test excavation to the northeast also were dated. For present analysis, we include only those samples from *in situ* deposits below the mound. We note, however, that the seven additional dates from mound fill and test excavation samples are not appreciably different from the ones included here.

Most recently, six other *Acropora* coral abraders were submitted to Weisler and Zhao for U/Th dating at the Centre for Microscopy and Microanalysis, University of Queensland. These were strategically selected from Lapita age strata from the Ha‘ateiho (Tongatapu), Tongoleleka (n=2, Ha‘apai), Vaipuna (Ha‘apai), Faleloa (Ha‘apai) and Ofu (Vava‘u) sites. U/Th dating protocols and methods are consistent with those previously applied to Nukuleka coral samples [13]. Close inspection of the coral abraders by Weisler documented that the fine sculptural features, such as verrucae, were sharp to the touch and well preserved. The samples, thus, were obtained from live corals prised from the reef with no in-built age in the dated artifacts. Furthermore, samples were judged pristine in the pretreatment phase by Zhao with limited if any evidence of diagenetic alteration. A double dating protocol as previously applied to some of the Nukuleka specimens [13] was not undertaken as a result. Samples were dated on a NuPlasma multiple collector inductively couple plasma mass spectrometer (MC-ICP-MS). The ages were calculated using Isoplot EX 3.0 program [14] with decay constants from Cheng et al [15].

**References**

1. Burley DV, Nelson E & Shutler R (1999) A radiocarbon chronology for the Eastern Lapita Frontier in Tonga. *Archaeology in Oceania* 34: 59-72.

2. Burley DV, Dickinson WR, Barton A & Shutler R (2001) Lapita on the periphery: New data on old problems in the Kingdom of Tonga. *Archaeology in Oceania* 36(2): 88-103.

3. Burley DV & Connaughton SP (2007) First Lapita settlement and its chronology in Vava‘u, Kingdom of Tonga. *Radiocarbon* 49(1): 131-137

4. Burley DV, Barton A, Dickinson WR, Connaughton SP & and Taché K (2010) Nukuleka as a founder colony for west Polynesian settlement: New insights from recent excavations. *Journal of Pacific Archaeology* 1: 128-144.

5. Petchey F & Clark G (2011) Tongatapu hardwater: Investigation into the ^14^C marine reservoir offset in lagoon, reef and open ocean environments of a limestone island. *Quaternary Geochronology* 6: 539-549.

6. Steadman DW, Pregill G & Burley DV (2002) Rapid prehistoric extinction of iguanas and birds in Polynesia. *PNAS* 99(6): 3673-3677.

7. Fisher R, Grant T & Harlow P (2012) *Brachylophus bulabula*. . IUCN Red List of Threatened Species. Version 2013.1 <http://www.iucnredlist.org/details/174471/0>.

8. Pregill GK & Steadman DW (2004) South Pacific iguanas: Human impacts and a new species. *Journal of Herpetology* 38(1): 15-21.

9. Kirch PV & Sharp WD (2005) Coral ^230^ Th dating of the imposition of a ritual control hierarchy in precontact Hawai‘i. *Science* 307: 102-104.

10. Weisler MI, Collerson KD, Feng Y, Zhao J-x, & Yu K (2006) Thoruim-230 coral chronology of a late prehistoric Hawai‘ian chiefdom. *Journal of Archaeological Science* 33: 273-282.

11. Weisler MI, Hua Q & Zhao J-x (2009) Late Holocene ^14^C marine reservoir corrections for Hawai‘i derived from U-series dated archaeological coral. *Radiocarbon* 51(3):955-968.

12. Sharp WD, Kahn JG, Polito CM & Kirch PV, (2010) Rapid evolution of ritual architecture in central Polynesia indicated by precise 230 Th/U coral dating. *PNAS* 107(30): 13234-13239

13. Burley DV, Weisler M & Zhao J-x. (2012) High Precision U/Th Dating of First Polynesian Settlement. *PLoS ONE* 7(11): e48769. doi:10.1371/journal.pone.0048769.

14. Ludwig KR (2003) Users Manual for Isoplot/Ex version 3.0: A Geochronological Toolkit for Microsoft Excel. In Berkeley Geochronology Centre Special Publication No.3

15. Cheng H, Edwards RL, Hoff J, Gallup CD, Richards DA, Asmerom Y (2000) The half-lives of uranium-234 and thorium-230. *Chemical Geology* 169(1-2): 17-33.

**16. Hogg A**, Hua Q, Blackwell P, Niu M, Buck C, Guilderson T et al. (2013) SHCal13 southern hemisphere calibration, 0-50,000 years cal BP. *Radiocarbon* 55(4):1889–1903.
